# Role of the Spike Glycoprotein of Human Middle East Respiratory Syndrome Coronavirus (MERS-CoV) in Virus Entry and Syncytia Formation

**DOI:** 10.1371/journal.pone.0076469

**Published:** 2013-10-03

**Authors:** Zhaohui Qian, Samuel R. Dominguez, Kathryn V. Holmes

**Affiliations:** 1 Department of Microbiology, University of Colorado School of Medicine, Aurora, Colorado, United States of America; 2 Institute of Pathogen Biology, Chinese Academy of Medical Sciences and Peking Union Medical College, Beijing, China; 3 Department of Pediatrics, University of Colorado School of Medicine, Aurora, Colorado, United States of America; Northeast Agricultural University, China

## Abstract

Little is known about the biology of the emerging human group c betacoronavirus, Middle East Respiratory Syndrome coronavirus (MERS-CoV). Because coronavirus spike glycoproteins (S) mediate virus entry, affect viral host range, and elicit neutralizing antibodies, analyzing the functions of MERS-CoV S protein is a high research priority. MERS-CoV S on lentivirus pseudovirions mediated entry into a variety of cell types including embryo cells from New World *Eptesicus fuscus* bats. Surprisingly, a polyclonal antibody to the S protein of MHV, a group a murine betacoronavirus, cross-reacted in immunoblots with the S2 domain of group c MERS-CoV spike protein. MERS pseudovirions released from 293T cells contained only uncleaved S, and pseudovirus entry was blocked by lysosomotropic reagents NH_4_Cl and bafilomycin and inhibitors of cathepsin L. However, when MERS pseudovirions with uncleaved S protein were adsorbed at 4°C to Vero E6 cells, brief trypsin treatment at neutral pH triggered virus entry at the plasma membrane and syncytia formation. When 293T cells producing MERS pseudotypes co-expressed serine proteases TMPRSS-2 or -4, large syncytia formed at neutral pH, and the pseudovirions produced were non-infectious and deficient in S protein. These experiments show that if S protein on MERS pseudovirions is uncleaved, then viruses enter by endocytosis in a cathepsin L-dependent manner, but if MERS-CoV S is cleaved, either during virus maturation by serine proteases or on pseudovirions by trypsin in extracellular fluids, then viruses enter at the plasma membrane at neutral pH and cause massive syncytia formation even in cells that express little or no MERS-CoV receptor. Thus, whether MERS-CoV enters cells within endosomes or at the plasma membrane depends upon the host cell type and tissue, and is determined by the location of host proteases that cleave the viral spike glycoprotein and activate membrane fusion.

## Introduction

Coronaviruses cause respiratory, enteric, renal and/or neurological disease in humans, many other mammals and birds. In 2002-03 a previously unknown coronavirus emerged from a wild animal reservoir to cause the SARS pandemic, with about 8,000 human cases and more than 770 deaths [1,2]. Previously, cross-species transmission from coronaviruses of bat and bovine origin had allowed human respiratory coronaviruses OC43, NL63 and 229E to become established in the human population worldwide [3–8]. In the Arabian Peninsula in 2012, another novel human CoV, now called Middle East Respiratory Syndrome Coronavirus (MERS-CoV), was isolated in Vero E6 cells from sputum from a fatal case of severe respiratory disease with kidney failure. Since then, MERS-CoV RNA has been detected by RT-PCR in over 70 patients with severe to moderate respiratory disease, 39 of whom have died [9,10]. Genome sequence analysis showed that MERS-CoV is a novel betacoronavirus in genogroup c, closely related to two prototype group c betacoronaviruses of Asian bats, BtCoV-HKU4 from a *Tylonycteris pachypus* bat and BtCoV-HKU5 from a *Pipistrellus abramus* bat [11], and to partial sequences of a group c betacoronavirus from a *Pipistrellus pipistrellus* bat in the Netherlands [12]. Recently group c betacoronaviruses were also detected in a *Nyctinomops laticaudatus* bat in Mexico [13], and *Nycyteris cf. gambiensis* bats in Ghana [14]. MERS-CoV, like SARS-CoV, is probably a zoonotic betacoronavirus that has spilled over into humans, directly or indirectly, from one of the species of bats that harbor group c betacoronaviruses or from other unknown animal reservoirs [13,15,16].

**Figure 1:**
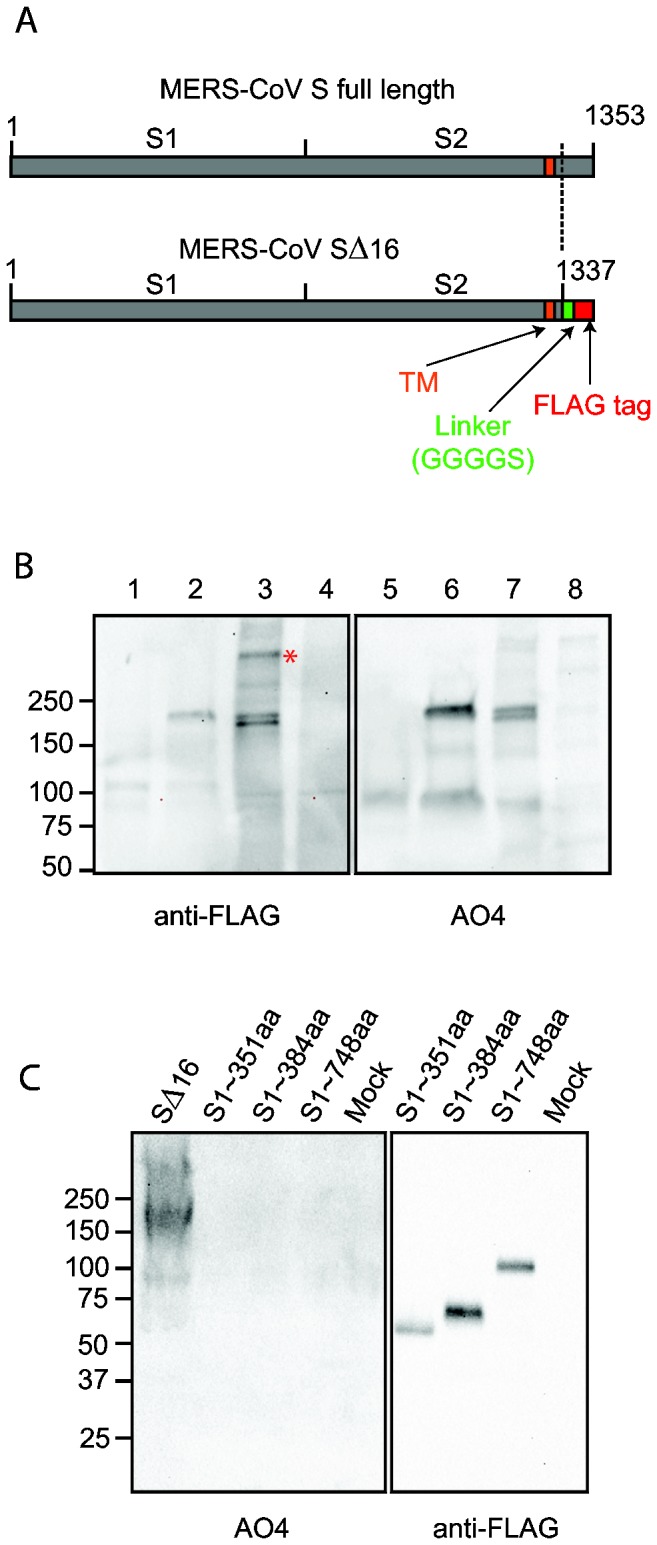
Incorporation of MERS-CoV SΔ16 protein into pseudovirions (A) Diagram of full length MERS-CoV S protein and MERS-CoV S∆16 protein with the C-terminal 16 amino acids substituted with a linker and FLAG tag. S1, virus attachment domain; S2, membrane fusion domain; TM, transmembrane domain. (B) Detection of uncleaved MERS-CoV SΔ16 protein in cells and pseudovirions. Lanes 1 and 5, control pseudovirions with no spike; lanes 2 and 6, MERS pseudovirions; lanes 3 and 7, lysate of 293T cells producing MERS pseudovirions; lanes 4 and 8, lysate of 293T cells producing control pseudovirions with no spike. Lanes 1 to 4 were blotted with anti-FLAG; and lanes 5 to 8, with polyclonal goat antibody AO4 to MHV-A59 S protein. * indicates possible trimer of MERS-CoV S protein in pseudovirions. In the pseudovirions made in 293T cells, the ~200 kDa S protein is uncleaved. (C) Identification of the domain of MERS-CoV S protein that is recognized by polyclonal antibody to MHV spike protein. Truncated MERS-CoV S proteins with c-terminal FLAG tags were harvested from the medium over transfected cells, analyzed by SDS-PAGE, and then blotted with anti-FLAG or AO4 antibody. A lysate of 293T cells expressing full length codon-optimized MERS SΔ16 is shown in Lane 1 as a positive control for recognition of MERS S by AO4.

The ~200 kDa spike glycoprotein (S) of coronaviruses is an important determinant of virus virulence, tissue tropism and host range. Trimers of S form the characteristic large spikes on the coronavirus envelope that bind to receptors, mediate membrane fusion, virus entry and syncytia formation, and elicit virus neutralizing antibodies. Coronavirus S proteins are Class I viral fusion proteins like the HIV envelope (env), influenza hemagglutinin (HA) and paramyxovirus fusion (F) glycoproteins [17], which typically require protease cleavage between the S1 and S2 domains (Figure 1A) to permit conformational changes in S2, activated by receptor binding and/or low pH, that mediate membrane fusion leading to virus entry and syncytia formation [3,17,18]. In different cell types and tissues, coronavirus S proteins may be cleaved by a variety of host proteases including furin, trypsin, human airway trypsin-like protease (HAT), transmembrane protease serine protease-2 (TMPRSS-2), TMPRSS-4, or cathepsins [18–22]. Functional analysis of MERS-CoV S glycoprotein is needed to identify susceptible cell types and host species that affect viral tissue tropism and host range, and to determine how various host proteases promote MERS-CoV virus entry and syncytia formation.

Identification of the receptor or receptors is an important first step in understanding the host range and tissue tropism of coronaviruses. Four receptor proteins for spike proteins of different coronaviruses are now known: murine carcinoembryonic antigen cell adhesion molecule 1a (mCEACAM1a) for mouse hepatitis virus (MHV) [23], a betacoronavirus in group a; aminopeptidase N (APN) for human coronavirus 229E (HCoV-229E) and several other alphacoronaviruses [24,25]; and angiotensin-converting enzyme 2 (ACE2) for both SARS-CoV, a betacoronavirus in group b and HCoV-NL63, an alphacoronavirus [26,27]. Raj and co-workers [28] recently demonstrated that MERS-CoV uses dipeptidyl peptidase 4 (DPP4) as a receptor. In contrast, S proteins of several group a betacoronaviruses including bovine coronavirus and hCoV-OC43 use sialic acid moieties as receptors [29,58]. We have used lentivirus pseudotypes with MERS-CoV spike glycoprotein to identify cells susceptible to infection with MERS-CoV and to study the role of MERS S protein in virus entry and syncytia formation.

## Results

### Expression of MERS-CoV spike (S) glycoprotein and incorporation into lentivirus pseudovirions

Expression of coronavirus S proteins on 293T cell membranes for incorporation into lentivirus pseudovirions can be enhanced by using codon-optimized spike cDNA and deleting an ER/Golgi retention motif and an endosomal recycling motif from the cytoplasmic tail of S [30–32]. Codon-optimized cDNA encoding S of MERS-CoV (derived from GenBank: AFS88936) [15], with the 16 C-terminal amino acids replaced by a linker, GGGGS, and a FLAG tag (here called MERS-CoV SΔ16) (Figure 1A) was expressed on 293T cell membranes and incorporated into envelopes of lentivirus pseudovirions. Immunoblotting of SDS-PAGE gels of the cell lysate (Figure 1B, lane 3) with anti-FLAG revealed two bands of MERS-CoV SΔ16 at about 200 kDa which probably reflect changes in glycosylation of SΔ16 during transport through the Golgi. Only the upper S band was incorporated into MERS pseudovirions (Figure 1B, lane 2). No protease cleavage products of the ~200kDa S protein were detected in transfected 293T cells or pseudovirions (Figure 1B). In marked contrast, the MERS lentivirus pseudovirions used to identify cells susceptible to entry of MERS-CoV in the Poehlmann laboratory [33], contained a high proportion of cleaved MERS-CoV S protein at about 100 kDa. This important difference in the MERS pseudovirions is likely due to differences between our 293T cells and those used in the Poehlmann laboratory. Surprisingly, when these MERS pseudovirions and cell lysates were blotted with polyclonal goat antibody AO4 to spikes purified from detergent-disrupted virions of MHV-A59, a betacoronavirus in group a, the MERS S protein bands were detected (Figure 1B). Immunoblotting of soluble, truncated MERS S proteins with C-terminal FLAG tags showed that the AO4 antibody did not recognize the S1 domain of MERS S (Figure 1C), so the cross-reactivity between these proteins from betacoronavirus groups a and c must lie within the S2 domain.

### MERS pseudovirions identify cell lines that have MERS-CoV receptor activity

**Figure 2:**
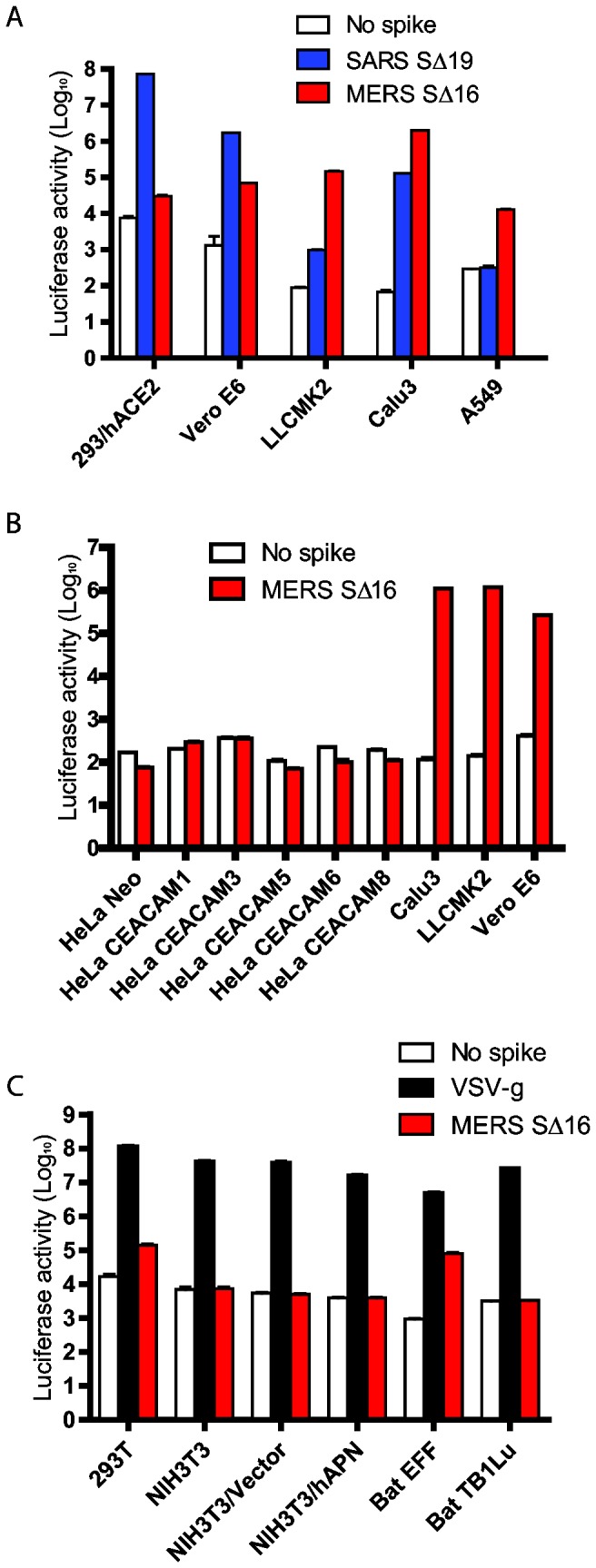
Entry into human, monkey and bat cells of pseudovirions with SARS-CoV, VSV or MERS-CoV glycoproteins. (A) Entry of SARS (blue), MERS-CoV (red) or no spike control (white) pseudovirions into 293/hACE2 (293 cells stably expressing hACE2, the SARS-CoV receptor), Vero E6, LLCMK2, Calu3, and A549 cell lines. Pseudovirus entry was quantitated by luciferase activity at 40 hrs post inoculation (pi). (B) Entry of MERS-CoV (red) or no spike control (white) pseudovirions into HeLa cells expressing 6 different human CEACAM proteins, Calu3, LLCMK2 and Vero E6 cells. (C) Entry of VSV (black), MERS-CoV (red) or no spike control (white) pseudovirions into different cell lines: 293T, NIH3T3, NIH3T3/Vector (transfected with empty vector), NIH3T3/hAPN (stably expressing human APN), bat EFF (embryo cells from *Eptesicus fuscus*), and bat TB1Lu (lung cells from *Tadarida brasiliensis*).

**Figure 3:**
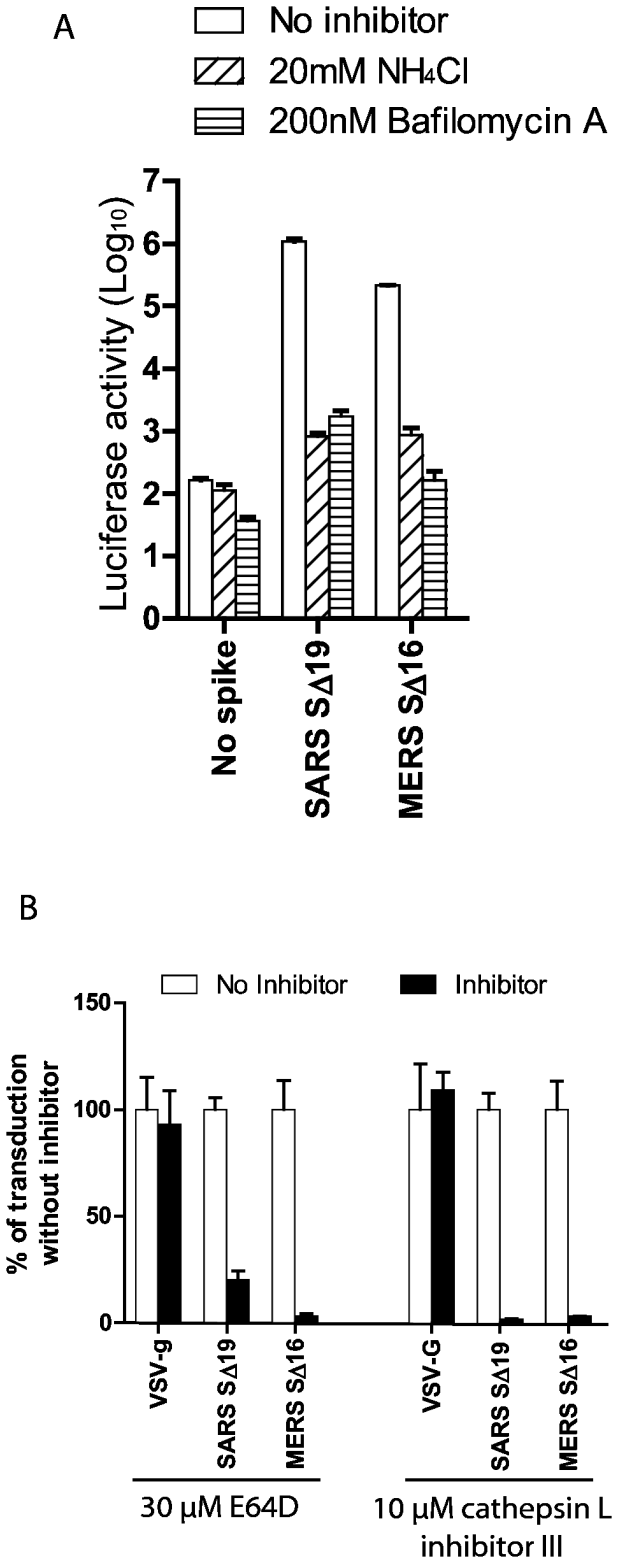
Inhibition of entry of MERS pseudovirions by lysosomotropic agents or cathepsin inhibitors. (A) Entry into Vero E6 cells of pseudovirions with SARS S protein, uncleaved MERS-CoV S protein or no spike control in the presence of 20mM NH_4_Cl (bars with rising stripes), 200nM bafilomycin A (bars with horizontal stripes), or medium alone (no inhibitor control, white bars). (B) Effects of cathepsin inhibitors on entry of pseudovirions with uncleaved MERS-CoV S protein, SARS S or VSV-g protein. Broad spectrum cathepsin inhibitor, E64D, or cathepsin L-specific inhibitor, cathepsin L inhibitor III (black bars) reduced entry of MERS and SARS pseudovirions, but did not inhibit entry of VSV pseudovirions relative to no inhibitor controls (white bars). Virus entry was quantitated by luciferase activity at 40hr pi.

Vero E6 and LLCMK2 monkey kidney cell lines are susceptible to infection with MERS-CoV virus and to SARS-CoV [10,34], and also susceptible to SARS pseudovirions and to MERS pseudovirions with uncleaved S protein (Figure 2A). Cell entry was quantitated by expression of the luciferase reporter gene in pseudovirus-transduced cells. Compared to control pseudovirions with no spike protein, MERS pseudovirions showed a 100 to 1,000 fold increase in luciferase activity in Vero E6 and LLCMK2 cells (Figure 2A and B), and SARS pseudovirions showed a 1,000 increase in luciferase activity in Vero E6 cells. Because the uncleaved MERS-CoV S protein mediated virus entry into Vero E6 and LLCMK2 cells, transduction by MERS pseudovirions was used to identify additional cell lines that express functional receptors for MERS-CoV [10,34]. MERS pseudovirions detected strong MERS-CoV receptor activity on the Calu3 line of human airway epithelial cells (Figure 2A and B), and weaker receptor activity on the A549 line of human alveolar basal epithelial cells (Figure 2A) as also shown by MERS-CoV infection [35]. Interestingly, the EFF embryo cell line from *Eptesicus fuscus* bats was susceptible to MERS pseudovirions, increasing luciferase activity by nearly 100-fold compared to the no spike control, but the TB1Lu lung cell line from *Tadarida brasiliensis* bats, murine fibroblasts and HeLa cells were not susceptible to MERS pseudovirions (Figure 2C). Expression of human ACE2 in 293T cells did not significantly increase susceptibility to MERS pseudovirions (Figure 3A), although as expected hACE2 greatly increased susceptibility of 293T cells to SARS pseudovirions (Figure 3A). Figure 3B and C show that neither human CEACAM1, or four related human CEACAM proteins or human APN functions as a receptor for MERS-CoV spike protein. These experiments confirm the observation that MERS-CoV does not use the receptor proteins known for other coronaviruses [33] or related human membrane proteins. Instead DPP4 is the principal receptor protein for MERS-CoV [28]. MERS pseudovirions induced a small but consistent 5 to 10-fold increase in luciferase activity in 293T human embryo kidney cells compared to the no spike control virus (Figure 2C), suggesting that our 293T cells expressed either a low level of DPP4, or an alternative but less efficient receptor, such as CD209L or LSECtin for SARS-CoV [36,37].

### Effects of inhibitors of acidification of endosomes on entry of MERS pseudovirions

To determine whether entry of MERS pseudovirions with uncleaved S protein required endocytosis and acidification in endosomes, the effects of ammonium chloride and bafilomycin A, lysosomotropic agents that inhibit the acidification of endosomes, were studied. In Vero E6 cells, 20mM NH_4_Cl inhibited entry of SARS pseudovirions by about 99.9% compared to entry of SARS pseudovirions without inhibitor, and NH_4_Cl also inhibited entry of MERS pseudovirions by about 99.6% (Figure 3A). Bafilomycin A specifically inhibits the vacuolar-type H+-ATPase that is required for acidification of lysosomes. Figure 3A shows that bafilomycin A inhibited entry of SARS pseudovirions into Vero E6 cells by 99.8% as previously reported [21,38], and also inhibited entry of MERS pseudovirions by more than 99.9% compared to MERS pseudovirions without inhibitor. In LLCMK2 cells, although bafilomycin A inhibited 99.7% of MERS-CoV S mediated entry, NH_4_Cl reduced MERS-CoV S-mediated entry only 6-fold (data not shown), suggesting that the inhibition of endosomal acidification by NH_4_Cl may be cell type dependent. These experiments show that MERS pseudovirions with uncleaved S protein can enter monkey kidney cells only by endocytosis.

### Entry of MERS pseudovirions in endosomes requires cleavage of S by cathepsin L

Cathepsins are a diverse group of acid-activated cysteine proteases located within endosomes and lysosomes. Cathepsin activity is essential for infection by several viruses that enter by the endosomal route, including reovirus [39], SARS-CoV [22], and Ebolavirus [40]. E64D, an inhibitor of the cysteine protease activities of cathepsins B, H, and L and calpain, reduced transduction of Vero E6 cells by SARS pseudovirions by 80% as previously reported (Figure 3B) [41]. Since cell entry mediated by VSV-g glycoprotein does not require protease activation [17], E64D treatment of Vero E6 (Figure 3B) and LLCMK2 cells (data not shown) did not inhibit entry of VSV pseudovirions. However, E64D decreased entry into Vero E6 cells of MERS pseudovirions with uncleaved S by 96.7% (Figure 3B) and LLCMK2 cells by 99.2% (data not shown). Thus, cleavage of MERS-CoV S protein by one of the cathepsins or calpain was required for triggering S-mediated membrane fusion and virus entry at low pH in endosomes. As previously reported [41], in Vero E6 cells 10 µM of cathepsin L inhibitor III, a specific and irreversible inhibitor of cathepsin L, significantly inhibited entry mediated by SARS S protein, but did not inhibit VSV-g-mediated entry (Figure 3B). Cathepsin L inhibitor III reduced entry into Vero E6 cells of MERS pseudovirions with uncleaved S protein by 97% relative to entry without inhibitor (Figure 3B), and similar results were seen in LLCMK2 cells (data not shown). Thus, MERS-CoV S protein on pseudovirions must be cleaved in endosomes by the acid-activated cysteine protease activity of cathepsin L to trigger receptor-dependent entry into Vero E6 and LLCMK2 cells.

### Trypsin cleavage of MERS-CoV S on pseudovirions adsorbed to receptors on the cell surface triggers virus entry at the plasma membrane at neutral pH

**Figure 4:**
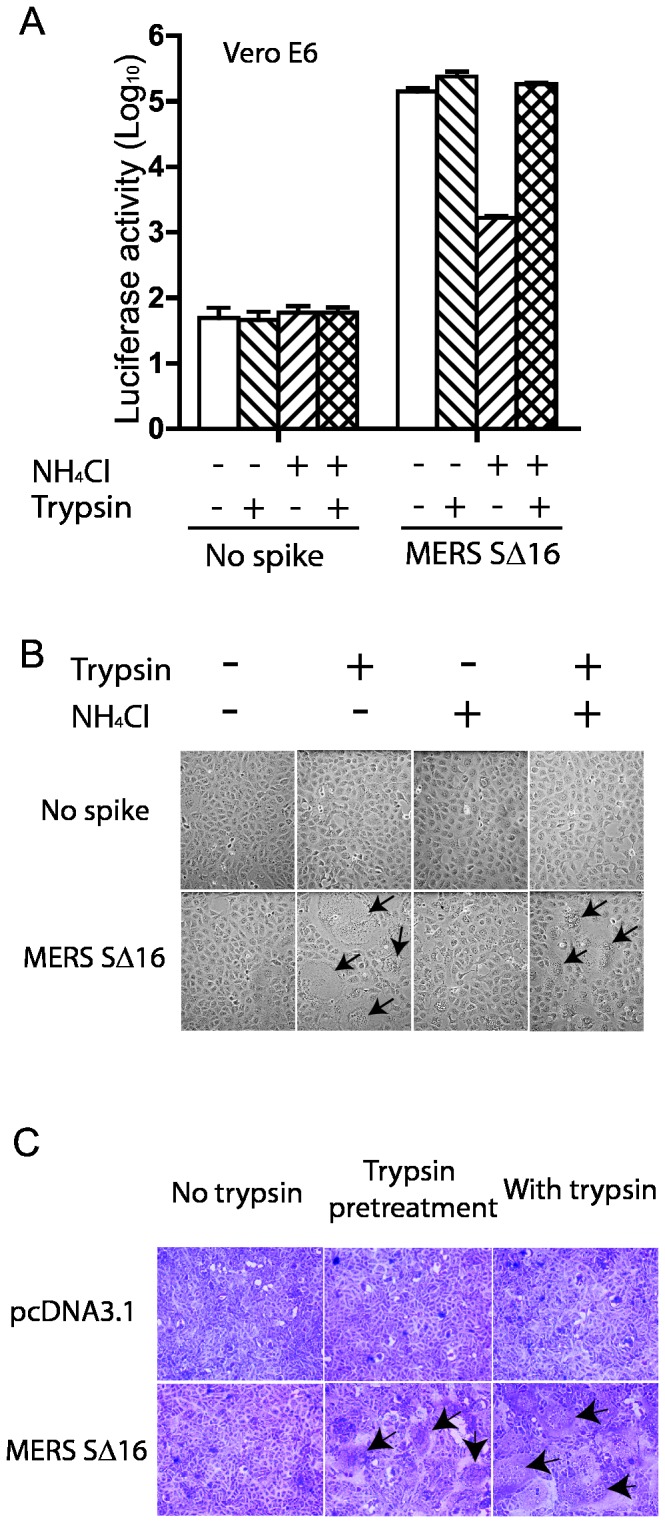
Trypsin activation of entry of MERS pseudovirions at the plasma membrane at neutral pH and MERS-CoV S-mediated syncytia formation. (A) MERS pseudovirions or no spike control pseudovirions were adsorbed on Vero E6 cells at 4°C, then briefly treated with trypsin or medium alone, followed by trypsin inhibitor in the presence or absence of NH_4_Cl to inhibit acidification of endosomes. Pseudovirus entry was quantitated by luciferase activity at 40 hrs pi. (B) In the same experiment, addition of trypsin activated S-mediated formation of scattered syncytia (black arrows) in Vero E6 cells with adsorbed MERS pseudovirions both in the presence and absence of NH_4_Cl at 40hr pi. (C) 293T cells stably transfected with plasmid encoding MERS-CoV SΔ16 protein or with no spike were briefly pre-treated with trypsin at 4°C, then with soybean trypsin inhibitor and then co-cultured with Vero E6 cells for 20 hr, or were co-cultured with Vero E6 cells for 20 hr in the continuous presence of trypsin. Syncytia formation required expression on the Vero E6 cells of the MERS-CoV receptor as well as trypsin cleavage of MERS-CoV S protein, but did not require acid pH.

SARS-CoV can enter susceptible cells at the plasma membrane, instead of by endocytosis, if virions adsorbed at 4°C to ACE2 on the cell membrane are treated with trypsin, then warmed to 37°C in the presence of an inhibitor of endosomal acidification [21]. Trypsin treatment at either 4°C or 37°C cleaved the S protein of MERS pseudovirions and generated a ~65kDa subunit in the S2 domain of the protein recognized by antibody to MHV-A59 S protein (Figure S1). MERS pseudovirions with uncleaved S protein were adsorbed at 4°C to cell surface receptors on Vero E6 cells in the presence of 20mM NH_4_Cl, and then the cells with bound virions were briefly treated with trypsin at pH 7.4 at room temperature to cleave the ~200 kDa S protein and activate its membrane fusing activity. Figures 3A and 4A show that NH_4_Cl strongly inhibited infection of Vero E6 cells by MERS pseudovirions with uncleaved S. However, trypsin treatment of the MERS pseudovirions bound at neutral pH and 4°C to the Vero E6 cell membrane triggered both virus entry at the plasma membrane and formation of small syncytia by 40 hours post inoculation (Figure 4A and B). Thus, receptor binding together with protease cleavage and activation of S at neutral pH was sufficient to trigger entry of MERS pseudovirions and syncytia formation. In this experiment membrane fusion did not depend upon synthesis of S protein, but syncytia formation was mediated by the cleaved S protein on pseudovirions adsorbed to virus receptor on the cell membrane. Although acidic pH is required to activate the cathepsin L activity that allows MERS pseudovirions to enter at endosomes, low pH is not required for the conformational changes in trypsin-cleaved MERS-CoV S protein that mediate entry at the plasma membrane.

### Trypsin cleavage of MERS-CoV S protein expressed on the cell surface induces syncytia formation in Vero E6 cells

293T cells expressing uncleaved MERS-CoV S protein or control cells stably transfected with the empty pcDNA3.1 vector were overlaid on monolayers of Vero E6 cells in the presence or absence of TPCK trypsin (Figure 4C). No syncytia formation was induced by 293T cells with empty vector or 293T cells expressing MERS-CoV SΔ16 without trypsin (Figure 4C), but addition of TPCK trypsin to the medium triggered formation of massive syncytia in the Vero E6 cells co-cultured for 20 hr with MERS-CoV S-expressing 293T cells (Figure 4C, arrows). Large syncytia were also formed after even a brief 20 minute trypsin pre-treatment at pH 7.4 and 4°C of 293T cells expressing MERS-CoV S protein, followed by incubation with a 5-fold excess of soybean trypsin inhibitor before layering the cells over confluent monolayers of Vero E6 cells and incubating at 37°C for 20 hours (Figure 4C, lower central panel, arrows). Thus, trypsin cleavage at neutral pH of MERS-CoV S protein on 293T cells triggered syncytia formation when S was bound to receptors on susceptible Vero E6 cells.

### Effects of TMPRSS-2 and -4 on entry of MERS pseudovirus and syncytia formation of 293T cells

**Figure 5:**
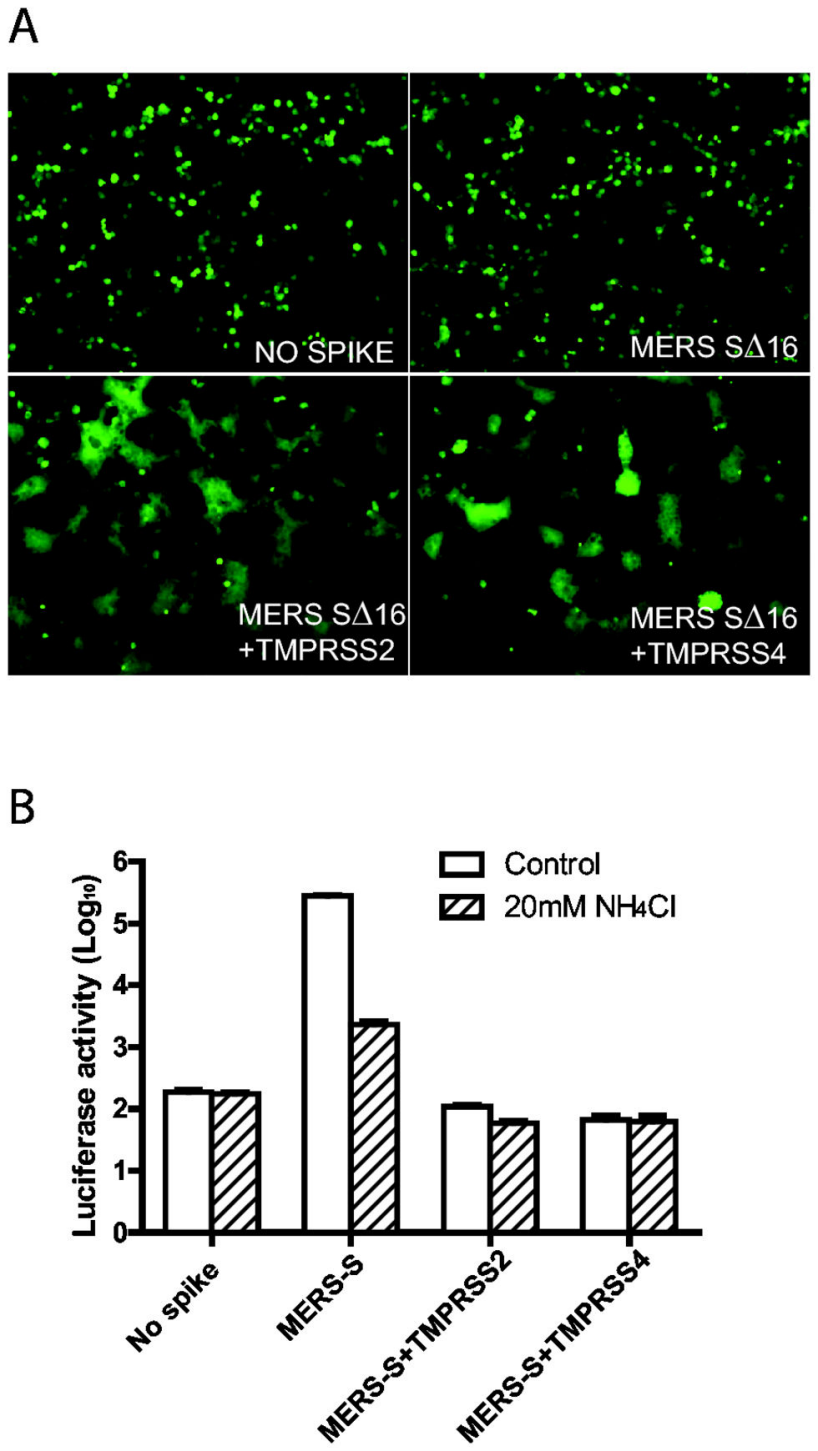
Effects of TMPRSS-2 and TMPRSS-4 on MERS-CoV S-mediated syncytia formation in 293T cells and MERS pseudovirus entry into Vero E6 cells. (A) Co-expression in 293T cells of the transmembrane serine proteases TMPRSS-2 or TMPRSS-4 as well as MERS pseudovirions induced syncytia formation in 293T cells expressing the GFP reporter gene, visualized 40hr after transfection. (B) MERS pseudovirions or no spike control pseudovirions released from the cells in Figure 5A were inoculated onto Vero E6 cells in the presence (striped bars) or absence (white bars) of 20mM NH_4_Cl to inhibit acidification of endosomes.

Type II transmembrane serine proteases, including TMPRSS-2 and TMPRSS-4, which like trypsin are expressed in the respiratory tract, play important roles in triggering entry of influenza A virus, human metapneumovirus and SARS betacoronavirus in group b [19,20,42–45]. We therefore transfected 293T cells with plasmids encoding TMPRSS-2 or -4, MERS-CoV SΔ16 protein, psPAX2 and pLenti-GFP-Luc and investigated whether S proteins on pseudovirions produced in these cells were cleaved and whether they could infect Vero E6 cells in the presence of NH_4_Cl. Surprisingly, the pseudovirion-producing 293T cells expressing either TMPRSS-2 or -4, formed large syncytia by 40 hrs after transfection (Figure 5A), but the MERS pseudovirions produced by these cells could not transduce Vero E6 cells in the presence or absence of NH_4_Cl (Figure 5B). In contrast, without TMPRSS-2 or -4, the 293T cells expressing uncleaved MERS-CoV SΔ16 did not form syncytia, and pseudovirions that they produced efficiently infected Vero E6 cells, but virus entry was inhibited by NH_4_Cl (Figure 5B). Immunoblots with antibody AO4 to MHV S or anti-FLAG (data not shown) revealed that the MERS pseudovirions produced in 293T cells expressing TMPRSS-2 or -4 contained little or no immunoreactive S protein or fragments of S.

## Discussion

Although the novel group c betacoronavirus MERS-CoV is highly virulent in humans and can infect cells from several different species, including humans, monkeys, pigs, and some species of bats [10,16,34], little is known about the biology of this virus. Because the spike glycoprotein is essential for coronavirus entry, elucidating the functions of the MERS-CoV spike can provide valuable insight into the pathogenesis of MERS-CoV, and suggest potential therapeutic interventions. Here we used lentivirus pseudovirions with MERS-CoV spike protein to study S-mediated cell entry at Biosafety Level 2.

We found that MERS pseudovirions, like infectious MERS-CoV virions [10,34], readily infected the Vero E6 and LLCMK2 lines of monkey kidney cells, several human respiratory epithelial cell lines, and embryo cells from *Eptesicus fuscus* bats. Others have also recently demonstrated that human respiratory tract cells, and also primary human bronchus and alveolar cells are susceptible to MERS-CoV in accord with the severe respiratory disease in MERS patients [10,33,35,46]. Muller et al. [34] recently reported that MERS-CoV can infect cells from four genera of Old World bats, *Rousettus, Rhinolophus, Pipistrellus*, and *Myotis*, and one New World genus, *Carollia*. We found that MERS pseudovirions could also infect cells from one New World bat, *E. fuscus*, but not from another, *T. brasiliensis*. The ability of MERS-CoV to infect cells from multiple mammalian species directly and without adaptation [47], including a diverse array of both Old World and New World bats, suggests that the receptor for MERS-CoV, DPP4 [28], is broadly conserved among many species, an important property of many emerging viruses [47,48]. *E. fuscus* bats, commonly known as big brown bats, are the bats most commonly encountered by humans in North America, and they are a reservoir for an alphacoronavirus [49,50]. It will be important to learn whether these New World bats are susceptible to MERS-CoV or related group c betacoronaviruses. The recent detection in *N. laticaudatus* bats in Mexico of a group c betacoronavirus with 96% similarity to MERS-CoV [13], coupled with the diverse array of alphacoronaviruses previously discovered in North American bats [49–52], justify increased surveillance to identify additional species of New World bats that may also harbor group c betacoronaviruses like MERS-CoV or other coronaviruses with the potential to cause severe disease in humans.

MERS-CoV is a betacoronavirus in group c, and we were surprised that its S protein was recognized in immunoblots by a polyclonal antibody to the spike protein of MHV-A59, a group a betacoronavirus. The cross-reactive epitope(s) was mapped to the S2 domain, which is more highly conserved than the S1 domain of betacoronaviruses. Chan et al. [33,53] found that MERS-CoV S protein was recognized in immunofluorescence and *in vitro* neutralization assays by sera of some convalescent SARS patients, and suggested, based on bioinformatics, that epitope(s) in S2 could account for the observed serological cross-reactivity. These observations that the S protein of MERS-CoV, a group c betacoronavirus, contains cross-reacting epitope(s) with S proteins of both some group b (SARS-CoV) and group a (MHV) betacoronaviruses, indicate that serological studies may not accurately distinguish between different phylogenetic groups of betacoronaviruses. Identification and characterization of the cross-reacting epitope(s) is an important research priority to show whether there is a common epitope in S that could be used as an immunogen to vaccinate against all betacoronaviruses.

Enveloped viruses infect cells by fusion of the viral envelope with host cell membranes, a process mediated by a series of conformational changes in the viral fusion protein that are regulated by receptor binding, protease activation, and/or pH [17]. The classes of viral fusion proteins are determined based on their structures and conformational changes during membrane fusion. Most class I viral fusion proteins require proteolytic cleavage upstream of the hydrophobic fusion peptide in the viral spike protein to enable these conformational changes to occur, as well as subsequent steps that trigger membrane fusion including either binding to receptor like HIV gp120, low pH in endosomes like influenza HA, or both like avian sarcoma leukosis virus (ASLV) [9,17,54–56]. Coronaviruses in different phylogenetic groups differ in the sequence of steps leading to virus entry [57,58]. The S protein on virions of group a betacoronavirus MHV-A59 requires protease activation--either by furin during virus maturation [18], by trypsin or other serine proteases in extracellular fluids before either receptor binding, or by cathepsin in endosomes at acidic pH—to trigger the conformational changes that lead to membrane fusion and virus entry [59]. In contrast, virions of group b betacoronavirus SARS-CoV that contain uncleaved S [22], first bind to the viral receptor protein, ACE2, and are endocytosed, and then S is cleaved within endosomal vesicles by acid-dependent cathepsin L enabling the conformational changes in S that lead to virus entry [21,22,60].

Here we analyzed the steps needed to trigger conformational changes in MERS-CoV S and their roles in virus entry and syncytia formation. In our laboratory, MERS pseudovirions released by 293T cells contained only uncleaved S protein, and, as for most coronaviruses, cleavage between S1 and S2 was necessary to enable its membrane fusing activity. The MERS pseudovirions bound to receptors on susceptible cells and were endocytized, and within the endosomes cleavage of S by the acid-dependent cysteine protease cathepsin L mediated virus entry. Gierer et al [33] reached similar conclusions using MERS pseudovirions that, unlike ours, contained more cleaved than uncleaved S protein, although both labs had made the pseudovirions in 293T cells. Gierer et al. [33] showed that batches of 293T cells differ markedly in expression of the MERS-CoV receptor and susceptibility to transduction by MERS pseudovirions. In our laboratory, the 293T cells showed minimal susceptibility to transduction with MERS-CoV pseudovirions with uncleaved S protein.

In addition to entry by endocytosis, we showed that, like SARS-CoV [21,22], MERS pseudovirions could enter susceptible Vero E6 cells at the plasma membrane if virions were first bound to cell surface receptors at 4°C at neutral pH in the presence of NH_4_Cl to inhibit acidification of endosomes, and also treated briefly at room temperature with trypsin to cleave the viral S protein. Upon warming to 37°C at neutral pH, the MERS pseudovirions fused with the plasma membrane and transduced the cells. Thus, MERS S protein does not require acidification to mediate virus entry, and the acidification required for endosomal entry [33] was required to activate the protease activity of cathepsin. Although treatment of coronavirus virions or pseudovirions with proteases can activate virus entry, it may also make them lose infectivity if the cleaved S1/S2 heterodimer dissociates before receptor binding. We found that MERS pseudovirions released from cells expressing TMPRSS-2 contained reduced amounts of S protein and had lost the ability to transduce susceptible cells. We postulate that the MERS pseudovirions that contain large amounts of cleaved S protein, detected by a c-terminal tag [33,53], could not enter cells at the plasma membrane because S1 may have dissociated from the cleaved spikes on virions. Development of antibodies specific for the S1 domain of MERS S protein are needed to test this hypothesis.

Coronavirus S proteins expressed on cell membranes can trigger receptor-dependent syncytia formation if the membrane-bound S protein is cleaved within the infected cells by furin or other proteases. MERS-CoV infection of Calu-3 and Caco-2 cell lines induced syncytia formation [35]. Our 293T cells were only minimally susceptible to entry of MERS-pseudovirions, and did not form syncytia when producing MERS pseudovirions with uncleaved S. However, when 293T cells expressing MERS-CoV SΔ16 protein were co-cultured in the presence of trypsin with Vero E6 cells that express the MERS-CoV receptor, enormous syncytia formed. These observations suggest that in tissues such as the lung, where trypsin, TMPRSS-2 or -4 and HAT and other serine proteases are available, MERS-CoV virus infection might spread directly from cell to cell by S-mediated, receptor-dependent syncytia formation, potentially escaping from virus-neutralizing antibodies, as do other syncytia-forming viruses such as respiratory syncytial virus, parainfluenza viruses, and measles. It will be important to learn whether syncytia are formed in lungs or other tissues of MERS-CoV patients or animal models of MERS-CoV.

Some coronavirus S proteins can also trigger receptor-independent syncytia formation [61,62]. When S proteins expressed on the plasma membrane are cleaved, the S1 domain can detach from the spike, exposing the hydrophobic fusion peptide of the membrane-anchored S2 domain that can directly induce fusion with any nearby cell membranes or lipid bilayers even if they lack receptors. This “receptor-independent spread (RIS)” allows an infected cell to fuse with adjacent non-infected, receptor-negative cells that can, in turn, produce virus and fuse with additional receptor-negative cells. RIS activity depends on the stability of S1/S2 interactions, and low stability of S1/S2 heterodimers correlates with rapid spread of infection through tissues that express little receptor protein [63,64]. We found that transmembrane serine proteases TMPRSS-2 and -4 could activate the syncytia forming activity of MERS-CoV SΔ16 protein expressed in 293T cells as these proteases do for Class 1 fusion proteins of other respiratory viruses including influenza, SARS-CoV and human metapneumovirus [19,20,42–45]. We were surprised that 293T cells, which express very little MERS-CoV receptor, were so extensively fused, and we hypothesize that this syncytia formation may be due to RIS.

Due to its high case fatality rate, therapeutic interventions for MERS-CoV are urgently needed. Pooled purified human immunoglobulin containing neutralizing antibody has been used to treat a variety of infectious diseases. Not surprisingly, since MERS-CoV is an emerging pathogen, we found that human immunoglobulin from the USA could not neutralize the infectivity of MERS pseudovirions (data not shown). However, sera from patients infected with either SARS-CoV or MERS-CoV contain antibodies that can neutralize MERS-CoV [33,53]. Most neutralizing antibodies would likely target the receptor-binding S1 domain of MERS-CoV S, which is less conserved than the S2 domain and can mutate, likely generating antibody escape mutants [65]. Here we showed that a polyclonal antibody to the S protein of MHV, a group a betacoronavirus, cross reacts with the S2 domain of MERS S protein in immunoblots. As we and others have proposed for SARS-CoVs [66,67], the more highly conserved S2 domain, and especially its C-terminal heptad repeat (HRC) region, can be important targets for blocking conformational changes in S, inhibiting syncytia formation and virus entry, and also eliciting neutralizing antibodies. If MERS-CoV virions made in the lung have uncleaved S protein and must therefore enter cells through endocytosis, then inhibitory HRC peptides, or neutralizing antibodies to HRC might not penetrate into the endosomes to prevent virus entry. However, MERS-CoV virions in the lung likely have cleaved S protein due to lung proteases, so that virus entry at the plasma membrane might be inhibited by HRC-targeted peptides or antibodies.

In summary, we demonstrated that, similar to SARS-CoV, cleavage of MERS-CoV-S protein by trypsin, TMPRSS2 or -4 or cathepsin L is required to activate the membrane fusion activity of S, leading to virus entry and syncytia formation, and that the location of the protease determines whether virus enters via endocytosis or by fusion at the plasma membrane. MERS-CoV S-mediated binding and entry mechanisms and protease triggering of conformational changes required for MERS-CoV-S virus entry and syncytia formation present potential targets for development of drugs or vaccines against this newly emerging and lethal group c human betacoronavirus.

## Materials and Methods

### Constructs and plasmids

Codon-optimized cDNA encoding the spike glycoprotein of MERS-CoV [15] was synthesized with the c-terminal 16 amino acids replaced with a GGGGS linker and a FLAG tag (GenScript, Piscataway, NJ), and for eukaryotic expression was cloned into pcDNA3.1(+) (Invitrogen) between the *BamHI* and *NotI* sites. To make constructs for expression of truncated soluble MERS S aa1-351, S aa1-384, and S aa1-748 proteins, PCR reactions were performed using the same forward primer AATGAAAAGCTTCACCATGATTCACTCCGTGTTCCTC pairing with the following reverse primers, for S aa1-351:

TAGTTTTCTAGAACTTCCGCCTCCACCATAA

 CTACAGTGGAGCTGGCT; for S aa1-384: 

TAGTTTTCTAGAACTTCCGCCTCCAC

CGTCGCACTCCACGCCTTCTGCC; or for S aa1-748 TAGTTTTCTAGAACTTC CGCCTCCACCTGGGGTCAGTGTGCTGGGGGT, and cloned into p3xFLAG-CMV 14 (Sigma, St Louis, MO) between HindIII and XbaI sites for expression. The VSV-g plasmid and lentiviral packaging plasmid, psPAX2, were obtained from Addgene (Cambridge, MA). The lentiviral reporter plasmid, pLenti-GFP-Luc, which expresses green fluorescent protein (GFP) and luciferase, was kindly provided by Fang Li, Duke University [68]

### Cell lines

The Vero E6 line of African green monkey kidney cells, the 293T line of human embryonic kidney cells transformed with SV40 large T antigen, the Calu 3 line of human airway epithelial cells, the A549 line of human alveolar epithelial cells, and the TB1Lu lung cell line from *T. brasiliensis* bats were obtained from ATCC (Manassas, VA). Hela cells stably expressing recombinant human CEACAM proteins and the control Hela cell line containing the empty vector were kindly provided by Scott Gray-Owen, University of Toronto [69]. The bat EFF cells were prepared by macerating mid-gestation fetuses of *Eptesicus fuscus* bats, briefly trypsinizing the cells, and plating them for expansion. Cells were passaged twice, frozen, and kindly provided by Richard Bowen, Colorado State University. Isolation of the EFF cells was conducted under approval 03-096A from the Colorado State University IACUC. Murine NIH3T3 cells stably expressing recombinant human aminopeptidase N (hAPN) or control cells with empty vector were previously described [70]. These cell lines were maintained in Dulbecco’s MEM with 10% fetal bovine serum (FBS) and 2% penicillin, streptomycin, and Fugizone (PSF) (Life Technologies Inc, Grand Island, NY). The LLC-MK2 line of rhesus monkey kidney cells from ATCC CCL-7 was maintained in Opti-MEM1 (Life Technologies Inc, Grand Island, NY) with 10% FBS and 2% PSF.

### Production of MERS pseudovirions and spike-mediated virus entry

Pseudotyped lentiviruses were produced as described previously [71] with minor modifications. Briefly, plasmids that encode viral spike glycoproteins MERS-CoV S∆16, SARS S∆19, or VSV-g were co-transfected into 293T cells with psPAX2 and pLenti-GFP-Luc using polyetherimide (PEI) (Polyscience Inc, Warrington, PA). Forty to 60 hr later the supernatant media containing pseudovirions were centrifuged at 800g for 5min to remove debris, and passed through a 0.45-µm filter. To quantitate entry of pseudovirions into different cell types, 250µl of pseudovirions with 8 µg/ml of polybrene (Sigma) was inoculated onto cells in 24-well plates, incubated overnight at 37°C, and cells were fed with fresh medium. At 40hr post inoculation (pi) cells were lysed at room temperature with 120µl of medium with an equal volume of Steady-glo (Promega, Madison, WI). Transduction efficiency was monitored by quantitation of luciferase activity. Living cells transduced by pseudovirions were detected by GFP expression. For all experiments triplicate samples were analyzed, data are representative of two or more experiments, and the standard error is shown.

### Detection of viral glycoproteins in pseudovirions

Pseudovirions with MERS-CoV SΔ16, SARS SΔ19, or VSV-g glycoprotein or control pseudovirions with no spike were centrifuged through a 20% sucrose cushion at 30,000 rpm at 4°C for 3 h in a Beckman SW41 rotor [71]. Spike proteins in the virions were separated on 4-15% SDS PAGE, blotted to nitrocellulose and detected with mouse anti-FLAG antibody M2 (Sigma, St Louis, MO) for MERS-CoV SΔ16, or polyclonal goat antibody AO4 to purified spikes from detergent-disrupted MHV-59 virions [72], followed by horseradish peroxidase (HRP)-conjugated antibody to mouse or goat IgG, and visualized with Chemiluminescent Reagent Plus (PerkinElmer, Boston, MA).

### Inhibition of pseudovirion entry by lysosomotropic agents

Vero E6 cells or LLCMK2 cells were incubated for 1hr at 37°C with medium alone or medium containing either 20mM NH_4_Cl or 200nM bafilomycin A to inhibit acidification of endosomes, and then spin-inoculated with pseudovirions and no spike controls in the presence of either 20mM NH_4_Cl or 200nM bafilomycin A for 90 min at 1,200g at 4°C. At 40 hr pi cells were lysed and luciferase activity was quantitated as a measure of virus entry. In Figure 3, the luciferase activities of Vero E6 cells transduced with VSV-, SARS-, and MERS-CoVS-pseudovirions without endocytosis inhibitor were 1x10^6^, 1.5-2x10^6^, and 5-6x10^5^, respectively.

### Effects of trypsin on entry of pseudovirions adsorbed to cell membranes

Monolayers of Vero E6 or LLCMK2 cells were incubated with 20mM NH_4_Cl in medium containing 10% FBS for 1hr at 37°C, then shifted for 15min to 4°C with 40mM NH_4_Cl. Pseudovirions were adsorbed to cells at 4°C by spin-inoculation for 90 min at 1,200g. The virus inocula were removed and replaced with pre-warmed, serum-free DMEM with or without 20mM NH_4_Cl. After 15min at 37°C, the media were replaced with serum-free DMEM with or without 15 µg/ml of TPCK-trypsin at room temperature to activate the membrane fusing activity of the S protein on virions adsorbed to the plasma membrane. Trypsin activity was then inhibited by incubation for 15 min on ice with 75µg/ml of soybean trypsin inhibitor (Worthington Biochemical Corporation, Lakewood, NJ) in DMEM with 10% FBS, and cells were incubated overnight at 37°C, fed with fresh medium, and incubated at 37°C. At 40 hr pi, virus entry was quantitated by luciferase activity and cells were photographed to detect viral cytopathic effects.

### Effects of cathepsin inhibitors upon entry of MERS pseudovirions

Monolayers of Vero E6 cells or LLCMK2 cells were pre-incubated for 2 hrs at 37°C with either 30µM of E64D, that inhibits cathepsin B, H, L and calpain, or 10µM of specific cathepsin L inhibitor III (Millipore, Billerica, MA). Pseudovirions and no spike controls with or without cathepsin inhibitors were spin-inoculated onto the cells at 4°C, then incubated at 37°C for 5 hours with or without the endocytosis inhibitors. Cells were then incubated at 37°C without inhibitors, and at 40 hr pi, luciferase activity in lysed cells was determined.

### MERS-CoV S∆16-mediated syncytia formation

293T cells in 6-well plates were transfected using PEI with 3µg of either empty vector or MERS-CoV S∆16 plasmid. For brief trypsin pre-treatment, cells were lifted with 1mM EDTA in PBS and washed, then incubated on ice with 20 µg/ml of TPCK trypsin or control medium for 20 min. Typsin activity was then inhibited by incubation with a five-fold excess of soybean trypsin inhibitor for 15min. The trypsin-pretreated 293T cells and control cells were then layered over monolayers of Vero E6 cells and incubated for 20 hr. Syncytia formation was detected by phase contrast microscopy or by imaging fixed cells stained with crystal violet.

## Supporting Information

Figure S1 **Detection of trypsin-cleaved MERS-CoV S protein on pseudovirions by antibody to MHV S protein.** Pseudovirions containing either MERS-CoV S protein or no spike (control) were incubated with 20 µg/ml of trypsin either at 4°C or 37°C for 20 min. After digestion, glycoproteins on pseudovirions were analyzed by immunoblot using AO4 antibody to the S protein of murine betacoronavirus MHV-A59 that cross-reacts with MERS-CoV S protein. Lanes 1 to 3: No spike control; Lanes 4 to 6: MERS pseudovirions; Lanes 1 and 4: No trypsin; Lanes 2 and 5: trypsin at 4°C; Lane 3 and 5: trypsin at 37°C. The band at ~90kDa was seen in pseudovirions without spike, indicating that it is not due to MERS-CoV S protein. Uncleaved MERS-CoV S protein, ~200kDa, on pseudovirions is shown in lane 4, and trypsin treatment cleaved all of the S protein, and generated a subunit of ~65kDa that was recognized by AO4 antibody (Lanes 5 and 6).
(EPS)
